# Encephalitis in Immunocompromised vs Immunocompetent Patients: A Comparative Study

**DOI:** 10.1093/ofid/ofaf332

**Published:** 2025-06-11

**Authors:** Anna Kolchinski, Margaret Li, Ralph Habis, Paris Bean, Ashley N Heck, John C Probasco, Rodrigo Hasbun, Arun Venkatesan

**Affiliations:** Johns Hopkins Encephalitis Center, Department of Neurology, Johns Hopkins University, School of Medicine, Baltimore, Maryland, USA; Johns Hopkins Encephalitis Center, Department of Neurology, Johns Hopkins University, School of Medicine, Baltimore, Maryland, USA; Johns Hopkins Encephalitis Center, Department of Neurology, Johns Hopkins University, School of Medicine, Baltimore, Maryland, USA; Department of Medicine, Section of Infectious Disease, McGovern Medical School, UTHealth, Science Center, Houston, Texas, USA; Department of Medicine, Section of Infectious Disease, McGovern Medical School, UTHealth, Science Center, Houston, Texas, USA; Johns Hopkins Encephalitis Center, Department of Neurology, Johns Hopkins University, School of Medicine, Baltimore, Maryland, USA; Department of Medicine, Section of Infectious Disease, McGovern Medical School, UTHealth, Science Center, Houston, Texas, USA; Johns Hopkins Encephalitis Center, Department of Neurology, Johns Hopkins University, School of Medicine, Baltimore, Maryland, USA

**Keywords:** checkpoint, herpes simplex, outcomes, prognostic, varicella zoster

## Abstract

**Background:**

Encephalitis is characterized by brain parenchymal inflammation caused by infection or autoimmunity. There are limited recent data on how immunocompromised patients with encephalitis differ from the general encephalitis population.

**Methods:**

This retrospective study of 2 large medical institutions compares clinical characteristics and outcomes of immunocompromised and immunocompetent patients with all-cause encephalitis.

**Results:**

Of the 657 patients, 151 (23%) were immunocompromised. Immunocompromised patients were more likely to have an infectious etiology, comorbidities, inflammatory cerebrospinal fluid (CSF) profile, abnormal neuroimaging, and worse clinical outcomes as assessed by discharge Glasgow Outcome Scale (GOS) and in-hospital mortality (all *P* < .05). The most commonly identified etiologies in immunocompromised patients were herpes simplex virus (HSV) and varicella zoster virus. HSV accounted for similar proportions in the immunocompromised (18%) and immunocompetent (14%) groups, though it was more commonly associated with a CSF neutrophilia in the immunocompromised group (*P* = .001). Strikingly, >10% of immunocompromised patients with encephalitis had autoimmune causes, two-thirds of which were checkpoint inhibitor associated. Factors associated with poor GOS on discharge differed, with poorer outcomes in the infectious group associated with immunocompromised state and poorer outcomes in the autoimmune group associated with immunocompetent state.

**Conclusions:**

Immunocompromised patients with encephalitis have differing causes, atypical clinical presentations, higher in-hospital mortality, and distinct factors associated with poor outcome as compared with immunocompetent patients. While HSV and opportunistic infections cause encephalitis in the immunocompromised, the diagnosis of autoimmune encephalitis should also be considered and can be checkpoint inhibitor associated.

## Teaser

Immunocompromised patients with encephalitis have differing causes, atypical clinical presentations, higher in-hospital mortality, and distinct factors associated with poor outcome as compared to immunocompetent patients. While less common, the diagnosis of autoimmune encephalitis should be considered in immunocompromised individuals.

Encephalitis is a syndrome defined by inflammation of the brain parenchyma, often presenting with fever, seizures, altered mental status, focal neurological deficits, cerebrospinal fluid (CSF) pleocytosis, and neuroimaging abnormalities reflective of acute inflammation [1]. Etiologies can be divided into infectious causes—many of which are well known—and a growing list of autoimmune etiologies.

The broad range of symptoms—including neurologic, psychiatric, and systemic—can make diagnosis of a particular encephalitis subtype challenging in the general population, and even more so in the immunocompromised population, who may present atypically and have a broader differential upon presentation [2, 3]. Few works examine the differences between immunocompromised and immunocompetent patients with encephalitis, although infectious causes have been much more commonly described in the immunocompromised [4–6]. Identification of these differences can inform differential diagnoses for immunocompromised patients, first by identifying when encephalitis as a syndrome should be considered and then by guiding the differential toward either an infectious or autoimmune etiology, thereby impacting treatment decisions.

Additionally, the incidence and characteristics of autoimmune encephalitis in the immunocompromised have not been well defined, although cases have been reported [7]. While autoimmune encephalitis may be less common in this population due to a decreased inflammatory response, paraneoplastic autoimmune encephalitis is well recognized, and autoimmune encephalitis may occur in other settings of immunocompromised status.

The purpose of our study was to describe the differences in clinical presentation, etiology, and outcomes between immunocompromised and immunocompetent adults with encephalitis.

## METHODS

### Study Population

Patients aged 18 years or older with International Classification of Diseases, 9th Revision (ICD-9), discharge codes corresponding to encephalitis were identified from 2 hospital systems in Houston, Texas, between February 2005 and February 2023 and from the Johns Hopkins Hospital and Johns Hopkins Bayview Medical Center between June 1, 2006, and March 15, 2016. Patients from this ICD-9 code–based screen were then selected based on fulfilling International Encephalitis Consortium (IEC) or Graus criteria for encephalitis [1, 8]. Additional patients were prospectively enrolled from the Johns Hopkins Hospital and outpatient center between January 2016 and December 2022 based on IEC or Graus criteria. A total of 658 patients, 372 from Houston, Texas, and 286 from Baltimore, Maryland, were included in the study [1, 8]. Classification of encephalitis patients as infectious or autoimmune necessitated a specific pathogen or autoimmune syndrome to be identified. The Johns Hopkins and University of Texas Institutional Review Boards (IRBs) approved this study.

### Data Extraction and Definitions

Immunocompromised status was determined based on specific criteria from past medical history at the initial encounter, including being infected with the HIV (regardless of CD4 count due to unavailability of recent CD4 count for many patients), active malignancy defined as malignancy not in remission at the time of hospitalization, solid organ or bone marrow transplantation, receiving ≥20 mg of prednisone daily or equivalent for >1 month, receiving other immunocompromising therapies, or having congenital immunodeficiencies [9].

Other data were collected from the patient's electronic medical records on their first day of presentation. These include demographics, symptoms at onset, Glasgow Coma Scale (GCS) score, serum white blood cell count, and Charlson Comorbidity Index (CCI) dichotomized at >2. The first CSF, magnetic resonance imaging (MRI), and electroencephalogram results were collected from the first hospital encounter. The boundary between acute vs subacute to chronic disease course was defined as >6 days from symptom onset to presentation.

Outcome measures including length of hospital stay, Glasgow Outcome Scale (GOS) <4, and mortality were collected at the date of discharge or death.

### Statistical Analysis

First, we conducted a comparative analysis of demographics, clinical features, and outcomes between immunocompromised and immunocompetent patients in our cohort of all-cause encephalitis. We then performed a subgroup analysis focusing on patients with infectious and those with autoimmune encephalitis. Next, we conducted a subanalysis to examine the association between variables readily available at presentation with poor outcome (GOS <4) at discharge. We included prespecified clinical variables based on the literature and clinical judgment, namely age, fever, focal neurologic findings, new-onset seizures, headache, memory loss, abnormal movements, CCI, encephalitis type, CSF characteristics, and peripheral white blood cell count (WBC) >11 ^000/µL.^

All statistical analyses were performed using IBM SPSS Statistics, version 29.0. The Pearson chi-square or Fisher exact test was used to assess categorical variables. The independent-samples *t* test and Mann-Whitney *U* test were used when appropriate to assess continuous and ordinal variables.

## RESULTS

### Comparison of Prospective and Retrospective Recruitment Groups in the Johns Hopkins Cohort

The 124 patients in the Johns Hopkins retrospective group and the 162 patients in the Johns Hopkins prospective group were similar in terms of gender, age, illness severity as assessed by GCS and death during hospitalization, and cause of immunocompromised status (*P* > .05 for all). They did differ in terms of encephalitis etiology, with autoimmune encephalitis being more common in the prospective cohort (*P* < .001), likely due to increased recognition of autoimmune syndromes in recent years.

### Comparison of Clinical Characteristics and Outcomes Between Immunocompromised and Immunocompetent Patients in All-Cause Encephalitis

#### Demographics

In our cohort, 151 of 657 (23%) of patients were immunocompromised (Table 1). The most common causes of immunocompromised status were HIV diagnosis (38%), immunosuppressive medications (30%), active malignancy (25%), and solid organ or bone marrow transplant (6%) (Supplementary Table 1). There was no difference in gender or age at symptom onset between the 2 groups. However, patients in the immunocompromised group were more likely to have a CCI >2, with 122/151 (81%) of immunocompromised patients meeting this criterion, as compared with only 184/506 (36%) in the immunocompetent group (*P* < .001).

**Table 1.** Demographics, Clinical and Lab Findings, and Outcome Variables for Total Encephalitis Cohort

	Immunocompromised		*P* Value
	Yes	No	
Patients, No.	151	506	
Sex, No.			.604
Female	83	266	
Male	68	240	
Mean age of onset (SD), y	50.23 (15.4)	48.46 (19.3)	.244
Total CCI >2, No. (%)	122/151 (81)	184/506 (36)	<.001***
Encephalitis type, No. (%)			<.001***
Autoimmune	18 (12)	109 (26)	
Infectious	85 (57)	183 (36)	
Unknown	48 (31)	214 (42)	
Clinical and lab findings, No. (%)			
Fever	86/144 (60)	252/480 (53)	.127
New focal CNS findings	65/150 (43)	188/498 (38)	.219
Seizures	46/149 (31)	212/487 (44)	.006**
Headaches	72/136 (53)	235/437 (54)	.865
New sleep disturbance	30/136 (22)	96/466 (21)	.713
New memory loss	34/143 (24)	170/489 (35)	.013*
Abnormal movements	17/144 (12)	94/486 (19)	.037*
CSF glucose <45 mg/dL	48/148 (32)	80/487 (16)	<.001***
CSF WBC ≥5/mm^3^	87/141 (62)	378/492 (77)	<.001***
CSF WBC neutrophil % >50%	28/131 (21)	81/405 (20)	.734
CSF protein <50 mg/dL	113/147 (77)	326/482 (68)	.033*
Peripheral WBC >11 K/µL	41/150 (27)	161/473 (34)	.126
MRI abnormalities	86/119 (72)	233/420 (55)	.001***
Glasgow Coma Scale, median (IQR)	14.00 (3.00)	14.00 (4.00)	.493
Subacute to chronic onset	47/144 (33)	115/488 (24)	.028*
Outcomes			
Hospital length of stay, mean (SD)	19.95 (22.5)	18.69 (23.6)	.566
Glasgow Outcome Scale <4, No. (%)	94/135 (70)	216/450 (48)	<.001***
Death, No. (%)	21/151 (14)	30/503 (6)	.001**

Abbreviations: CCI, Charlson Comorbidity Index; CNS, central nervous system; CSF, cerebrospinal fluid; IQR, interquartile range; MRI, magnetic resonance imaging; WBC, white blood cell count.

*P < .05; **P < .01; ***P < .001 (all indicated in bold).

#### Etiology

**Figure 1.**
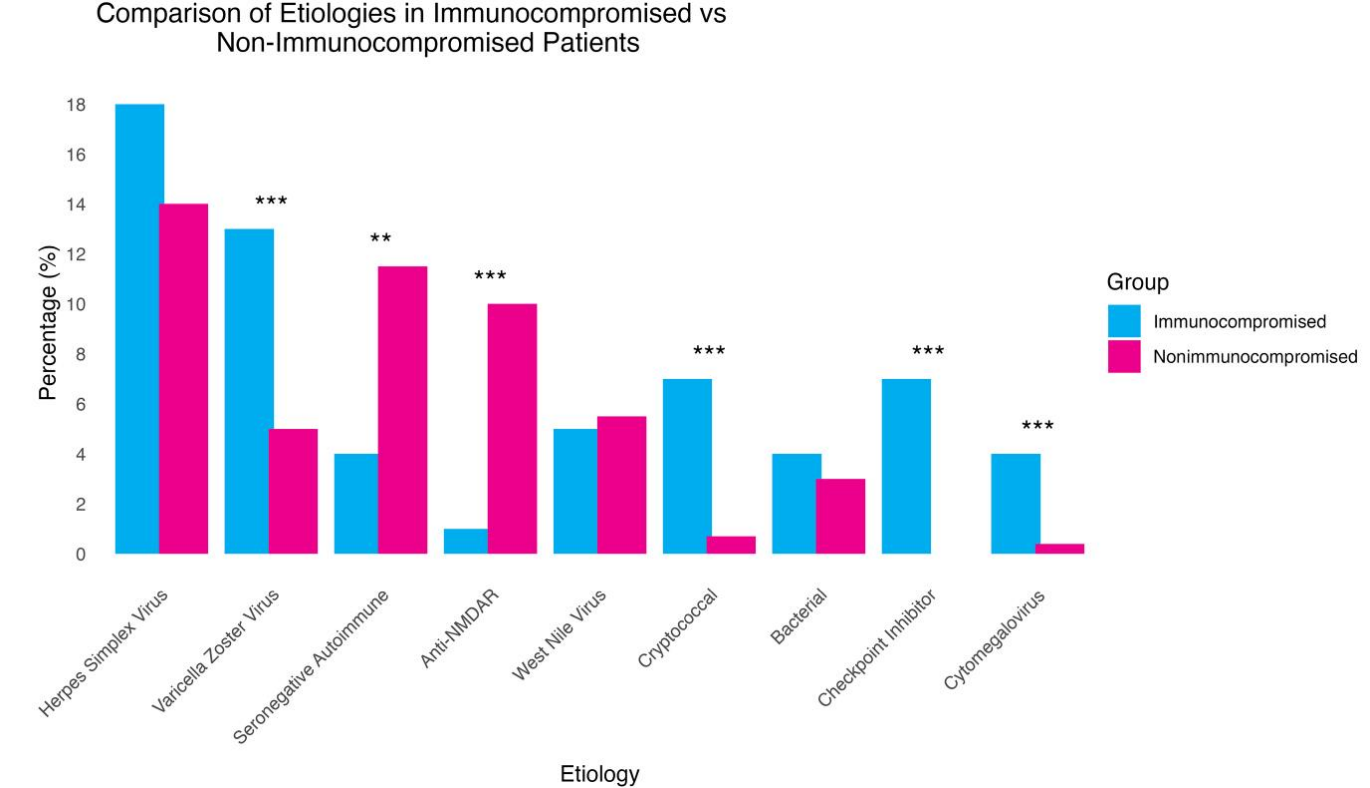
A comparison of encephalitis etiologies between immunocompromised and immunocompetent patients. *P < .05; **P < .01; ***P < .001. Abbreviation: NMDAR, N-methyl-D-aspartate receptor.

Immunocompromised individuals were more likely to develop infectious encephalitis (85/151 [57%]) as compared with immunocompetent individuals (183/506 [36.0%]; *P* < .001) and less commonly developed autoimmune encephalitis (18/151 [12%] vs 109/506 [26%] in immunocompetent; *P* < .001) (Table 1). As expected, the specific etiological profile differed between immunocompromised and immunocompetent individuals (Figure 1), although HSV was the most common etiology in both groups (27/151 [17.9%] immunocompromised vs 71/506 [14%] immunocompetent; *P* = .27) (Table 2). Among the immunocompromised cohort, additional common etiologies were varicella zoster virus (VZV) encephalitis (n = 19), checkpoint inhibitor–associated encephalitis (n = 11), cryptococcal encephalitis (n = 11), anti-N-methyl-D-aspartate receptor (NMDAR) encephalitis (n = 2), and West Nile virus (WNV) encephalitis (n = 7). In contrast, additional etiologies in the immunocompetent group included anti-NMDAR encephalitis (n = 53), WNV (n = 28), VZV (n = 25), Hashimoto's encephalopathy (n = 19), anti-leucine-rich glioma-inactivated 1 (LGI1) encephalitis (n = 13), and antiglutamic acid decarboxylase 65 (GAD65) encephalitis (n = 12) (Table 2).

**Table 2.** Comparison of Encephalitis Etiologies

Etiology:	Immunocompromised, No. (%)	Nonimmunocompromised, No. (%)	Significance
Herpes simplex virus	27/151 (18)	71/506 (14)	.244
Varicella zoster virus	19/151 (13)	25/506 (5)	<.001***
Cryptococcal	11/151 (7)	4/506 (0.7)	<.001***
West Nile virus	7/151 (5)	28/506 (5.5)	.666
Cytomegalovirus	6/151 (4)	2/506 (0.4)	<.001***
Anti-NMDAR	2/151 (1)	53/506 (10)	<.001***
Seronegative	6/151 (4)	58/506 (11)	.006**
Checkpoint inhibitor	11/151 (7)	0/506 (0)	<.001***
Bacterial	6/151 (4)	15/506 (3)	.536
Hashimoto's encephalopathy	0/151 (0)	19/506 (4)	.016*
Anti-LGI1	0/151 (0)	13/506 (3)	.047*
Anti-GAD65	0/151 (0)	12/506 (2)	.056

Abbreviations: GAD65, glutamic acid decarboxylase 65; LGI1, leucine-rich glioma-inactivated 1; NMDAR, N-methyl-D-aspartate receptor.

*P < .05; **P < .01; ***P < .001 (all indicated in bold).

#### Clinical Presentation

Immunocompromised patients were more likely than the immunocompetent group to have a subacute to chronic onset (47/144 [33%] vs 115/488 [24%]; *P* = .028), to have brain MRI abnormalities of any type (86/119 [72%] vs 233/420 [55%]; *P* < .001), and were more likely to have CSF glucose <45 mg/dL (48/148 [32%] vs 80/487 [16%]; *P* < .001). However, immunocompromised patients presented less frequently than immunocompetent patients with memory loss (34/143 [24%] vs 170/489 [35%]; *P* = .013), abnormal movements (17/144 [12%] vs 94/486 [19%]; *P* = .037), and seizures (46/149 [31%] vs 212/487 [44%]; *P* = .006). No differences were observed between the 2 groups regarding fever or peripheral WBC count (Table 1).

#### Hospital Course and Discharge Status

As shown in Table 1, immunocompromised patients had higher in-hospital mortality (21/151 [14%] vs 30/503 [6%]; *P* < .001) and more commonly had a Glasgow Outcome Scale <4 on discharge (94/135 [70%] vs 216/450 [48%]; *P* < .001).

#### Infectious Encephalitis Subgroup Analysis

Of the 268 patients with infectious encephalitis, 85 (32%) were immunocompromised (Supplementary Table 2). As in the entire cohort, immunocompromised infectious encephalitis patients were more likely to have a CCI >2 (70/85 [82%] vs 76/183 [43%]; *P* < .001).

Immunocompromised patients were less likely to have a CSF pleocytosis (51/81 [63%] vs 134/179 [75%]; *P* = .05) and more commonly had a CSF glucose <45 mg/dL (36/85 [42%] vs 35/180 [19%]; *P* < .001). While inpatient mortality did not differ, immunocompromised patients were more likely to have increased length of hospital stay (22.7 vs 14.1 days; *P* = .01) and a GOS <4 on discharge (56/78 [72%] vs 67/165 [41%]; *P* < .001).

We next examined the subgroup of patients with HSV encephalitis, comprised of 98 patients, 27 (27.6%) of whom were immunocompromised (Table 3). Aside from sleep disturbances, which were more commonly seen in the immunocompromised group (7/20 [35%] vs 8/67 [12%]; *P* = .017), other clinical signs and symptoms such as presence of fever, new-onset seizures, and memory loss did not differ between the 2 groups. However, brain MRI abnormalities were less commonly found in the immunocompromised group (13/21 [62%] vs 48/57 [84%]; *P* = .034). While the proportion of pleocytosis did not differ between the 2 groups, CSF neutrophilia, defined as a CSF WBC count in which >50% of cells are neutrophils, was more commonly seen in the immunocompromised group (8/26 [31%] vs 3/60 [5%]; *P* = .001). Hospital length of stay was increased in immunocompromised individuals, as was the proportion of individuals with GOS <4 at discharge (Table 3).

**Table 3.** Demographics, Clinical and Lab Findings, and Outcome Variables for Patients With Herpes Simplex Virus Encephalitis

	Immunocompromised		*P* Value
	Yes	No	
Patients, No.	27	71	
Sex, No.			.277
Female	17	36	
Male	10	35	
Mean age of onset (SD), y	57 (10.48)	54.23 (16.55)	.21
Total CCI >2, No. (%)	24/27 (89)	30/72 (42)	<.001***
Clinical and lab findings, No. (%)			
Fever	18/26 (69)	51/71 (72)	.802
New focal CNS findings	15/27 (56)	26/71 (37)	.09
Seizures	10/27 (37)	32/71 (35)	.473
Headaches	14/23 (61)	44/63 (70)	.432
New sleep disturbance	7/20 (35)	8/67 (12)	.017*
New memory loss	2/22 (9)	12/69 (17)	.347
Abnormal movements	4/25 (16)	8/70 (11)	.555
Subacute to chronic	1/23 (4)	10/68 (15)	.188
CSF WBC ≥5/mm^3^	18/27 (67)	56/69 (81)	.129
CSF WBC neutrophils % >50%	8/26 (31)	3/60 (5)	.001***
CSF protein <50 mg/dL	7/25 (28)	13/70 (19)	.321
CSF glucose <45 mg/dL	7/27 (26)	10/69 (14)	.187
Peripheral WBC >11 K/µL	6/27 (22)	13/69 (19)	.708
Glasgow Coma Scale, median (IQR)	13.00 (2.50)	14.00 (3.245)	.167
MRI abnormalities	13/21 (62)	48/57 (84)	.034*
Abnormal EEG	19/21 (90)	37/50 (74)	.121
Treatments, No. (%)			
Vancomycin^a^	22/27 (81)	42/71 (59)	.038*
Acyclovir	27/27 (100)	69/71 (97)	.378
Corticosteroids	17/27 (63)	19/70 (27)	.001***
Outcomes			
Hospital length of stay, mean (SD)	22 (20.5)	13.55 (13.1)	.01**
Glasgow Outcome Scale <4, No. (%)	21/25 (84)	23/57 (40)	<.001***
Death, No. (%)	5/27 (19)	16/71 (23)	.665

Abbreviations: CCI, Charlson Comorbidity Index; CNS, central nervous system; CSF, cerebrospinal fluid; EEG, electroencephalogram; HSV, herpes simplex virus; IQR, interquartile range; MRI, magnetic resonance imaging; WBC, white blood cell count.

*P < .05; **P < .01; ***P < .001 (all indicated in bold).

^a^Vancomycin included to highlight the high rate of empiric treatment of bacterial infection in patients ultimately found to have an HSV infection.

#### Autoimmune Encephalitis Subgroup

Among the 127 patients with autoimmune encephalitis, 18 (14%) were immunocompromised (Table 4). The immunocompromised group was older (mean [SD], 56.7 [15.6] vs 41.2 [21.9]; *P* = .002) and had a higher CCI >2 (15/18 [83%] vs 35/109 [32%]; *P* < .001). Two-thirds of cases (12/18) were associated with exposure to immune checkpoint inhibitors. Immunocompromised patients were less likely than immunocompetent patients to present with abnormal movements (4/18 [22%] vs 53/99 [54%]; *P* = .014) or seizures (5/18 [28%] vs 79/108 [73%]; *P* < .001). MRI abnormalities were more commonly observed in immunocompromised individuals (12/17 [71%] vs 46/104 [44%]; *P* = .044). Notably, the mean (SD) hospital length of stay was substantially lower in the immunocompromised cohort (15.9 [13.7] vs 33.0 [33.1] days; *P* < .001). However, no differences were seen between the 2 groups in terms of GOS <4 on discharge or in-hospital mortality (Table 4).

**Table 4.** Demographics, Clinical and Lab Findings, and Outcome Variables for Autoimmune Encephalitis Patients

	Immunocompromised		*P* Value
	Yes	No	
Patients, No.	18	109	
Sex, No.			.971
Female	9	55	
Male	9	54	
Mean age of onset (SD), y	56.72 (15.6)	41.22 (21.9)	.002**
Clinical and lab findings, No. (%)			
Fever	6/18 (33)	24/96 (25)	.461
New focal CNS findings	8/18 (44)	48/109 (44)	.974
Seizures	5/18 (28)	79/108 (73)	<.001***
Headaches	10/17 (59)	34/88 (39)	.123
New sleep disturbance	10/18 (56)	53/91 (58)	.833
New memory loss	10/18 (56)	60/104 (58)	.866
Abnormal movements	4/18 (22)	53/99 (54)	.014*
CSF WBC ≥5/mm^3^	10/15 (67)	77/105 (73)	.589
CSF WBC neutrophils % >50%	0/15 (0)	4/81 (5)	.379
CSF protein <50 mg/dL	6/17 (35)	67/102 (66)	.017*
CSF glucose <45 mg/dL	1/16 (6)	10/103 (10)	.657
Peripheral WBC >11 K/µL	3/18 (17)	31/101 (31)	.225
Glasgow Coma Scale, median (IQR)	14.00 (3.00)	14.00 (5.00)	.174
Total CCI >2	15/18 (83)	35/109 (32)	<.001***
MRI abnormalities	12/17 (71)	46/104 (44)	.044*
Abnormal EEG	13/17 (76)	79/103 (77)	.984
Subacute to chronic onset	6/18 (33)	26/105 (25)	.444
Outcomes			
Hospital length of stay, mean (SD)	15.94 (13.68)	32.98 (33.1)	<.001***
Glasgow Outcome Scale <4, No. (%)	9/16 (56)	57/89 (64)	.552
Death, No. (%)	1/18 (6)	4/108 (4)	.709

Abbreviations: CCI, Charlson Comorbidity Index; CNS, central nervous system; CSF, cerebrospinal fluid; EEG, electroencephalogram; IQR, interquartile range; MRI, magnetic resonance imaging; WBC, white blood cell count.

*P < .05; **P < .01; ***P < .001 (all indicated in bold).

### Factors Associated With Outcomes

We next examined the association between variables at presentation with poor outcome at discharge (Table 5). In immunocompetent individuals, older age (*P* = .002), presence of focal neurological findings (*P* = .009), presence of seizures (*P* = .004), presence of headaches (*P* = .007), abnormal movements (*P* < .001), and peripheral WBC >11 × 10^3^/µL (*P* = .03) were associated with poor outcome (GOS <4). In the immunocompromised cohort, on the other hand, only the presence of fever (*P* = .023) and seizures (*P* = .016) were factors associated with poor outcome. Factors associated with poor outcome also differed in several subgroups of patients with encephalitis (Supplementary Table 3).

**Table 5.** Glasgow Outcome Scale Predictor Variables

	Glasgow Outcome Scale		
Significant Predictors	GOS <4, No. (%)	GOS ≥4, No. (%)	*P* Value
Cohort 1: all encephalitis types, nonimmunocompromised			
Age >60 y	86/216 (40)	61/234 (26)	.002
New focal CNS findings at onset	89/211 (42)	70/231 (30)	.009
Seizures	103/208 (50)	82/229 (36)	.004
Headaches at onset	82/177 (46)	126/210 (60)	.007
Abnormal movements at onset	50/204 (25)	27/228 (12)	<.001
Type of encephalitis	Autoimmune: 57/216 (26) Infectious: 67/216 (31)	Autoimmune: 32/234 (14) Infectious: 98/234 (42)	.002
Peripheral WBC >11 K/µL	81/203 (40)	67/224 (30)	.03
Cohort 2: all encephalitis types, immunocompromised			
Fever at onset	59/89 (66)	18/40 (45)	.023
Seizures	35/92 (38)	7/41 (17)	.016

Abbreviations: CNS, central nervous system; GOS, Glasgow Outcome Scale; WBC, white blood cell.

## DISCUSSION

In this study of 657 adults with encephalitis from 2 academic medical centers, >20% of patients were immunocompromised, accounting for a substantial proportion of those with encephalitis. Key findings included (1) in contrast to opportunistic infections such as VZV, CMV, and cryptococcus, the proportion of patients with HSV encephalitis was similar among the immunocompromised and immunocompetent groups; (2) among HSV encephalitis patients, CSF neutrophilia was more commonly seen in the immunocompromised group; (3) among the immunocompromised population, while infections accounted for the largest proportion of cases, >10% were autoimmune in nature; (4) distinct factors were associated with poor outcome in immunocompromised, as compared with immunocompetent, patients.

Perhaps unsurprisingly, immunocompromised patients were more likely to be diagnosed with infectious encephalitis than immunocompetent individuals. However, while VZV was far more represented in the immunocompromised group, this was not the case for the closely related virus HSV. Our data underscore the notion that encephalitis associated with HSV, unlike VZV, should not generally be considered an opportunistic infection. The reasons for this difference remain unclear given the similarities in genomic structure and latent reservoirs among the 2 herpesviruses [10]. One potential explanation is that CNS HSV infection may be underdiagnosed in the immunocompromised, given the potential for atypical clinical presentations of HSV encephalitis, as well as other more subtle forms of CNS infection in immunocompromised individuals that may only become apparent on postmortem investigation [11].

Prior, smaller studies have found that immunocompromised hosts diagnosed with HSV encephalitis were less likely to have a CSF WBC pleocytosis, making diagnosis more challenging and necessitating the use of HSV PCR for confirmation even in the absence of pleocytosis [12, 13]. Indeed, a recent study showed that up to 25% of patients with HSV encephalitis may not have a pleocytosis on initial lumbar puncture [14]. While we did not find a difference in CSF WBC between immunocompromised and immunocompetent individuals with HSV encephalitis, we did observe that those who were immunocompromised were more likely to demonstrate CSF neutrophilia. While CSF neutrophils are commonly seen in bacterial infections and early in viral encephalitis, there is typically a rapid shift to lymphocytic predominance in viral encephalitis, aside from several notable exceptions including WNV and, in the immunocompromised, CMV infections [1, 15–17]. Previous studies in human patients with HSV encephalitis have shown increases in serum and CSF IL-8 for 14 hours after admission, supporting the role of neutrophil trafficking being most important early in the disease process [18]. Additionally, histopathological and murine model studies of HSV encephalitis have implicated neutrophils as a crucial mediator of viral load reduction before a lymphocytic response can be mounted [19, 20]. It is possible that the dampened ability of some immunocompromised patients to mount a lymphocytic response may necessitate prolonged neutrophilic involvement during HSV infection.

Autoimmune encephalitis was more common in the immunocompetent group and tended to occur in patients who presented with seizures, movement disorders, and memory deficits. Notably, however, >10% of our immunocompromised cohort had autoimmune encephalitis, underscoring the need to remain vigilant for patients with autoimmunity even in the setting of immunocompromising factors. Broadly, etiologies could be viewed as immune checkpoint–associated encephalitis vs others (including paraneoplastic encephalitis, traditional autoantibody-mediated encephalitis, and infection-associated autoantibody-mediated encephalitis). Overall, immunocompromised autoimmune encephalitis patients were less likely to present with features of typical autoimmune encephalitis, including seizures and movement disorders. This may in part be because two-thirds of the patients in the autoimmune encephalitis immunocompromised cohort had checkpoint inhibitor–associated disease. Additionally, while in our study we found an increased prevalence of MRI abnormalities in autoimmune encephalitis in the immunocompromised vs immunocompetent group, prior work on checkpoint inhibitor–associated encephalitis has shown a broad range of MRI findings, indicating the need for larger studies to establish the true prevalence of abnormalities [21, 22]. Our findings of atypical features in immunocompromised patients who developed autoimmune encephalitis, along with the reported poor sensitivity of laboratory testing for autoimmune encephalitis–related antibodies in patients on checkpoint inhibitor therapy, highlight the challenges in diagnosing autoimmune encephalitis in the setting of immunocompromised state [22, 23].

Previously reported poor prognostic factors in encephalitis in immunocompetent individuals include older age and major medical comorbidities [4, 24–29]. We found that older age, along with several distinct clinical characteristics including movement disorders and autoimmune rather than infectious etiology, was associated with lower GOS in our immunocompetent individuals. This contrasts with our immunocompromised group, where associated factors included fever and seizures. Notably, the poor outcomes in immunocompromised individuals appear to be driven largely by the subset of those with infectious encephalitis. In those with autoimmune encephalitis, immunocompromised state was not associated with GOS or with increased mortality, and in fact hospital length of stay was substantially lower than in the immunocompetent patients. This may reflect the natural history and potential reversibility of checkpoint inhibitor–associated encephalitis in the immunocompromised group. Indeed, prior case studies of checkpoint inhibitor–associated encephalitis in melanoma patients showed a very high and rapid response rate to corticosteroids in combination with checkpoint therapy discontinuation, in contrast to other forms of autoimmune encephalitis, which may be more refractory [23].

### Strengths, Limitations, and Future Directions

This study has several unique strengths, the most notable being that it is the largest study examining all-cause encephalitis in the immunocompromised population, and one in which comparisons can be readily made to immunocompetent individuals. Additionally, the inclusion of participants from 2 geographically different academic medical centers improves the generalizability of our results. A limitation of the study is that patients from Johns Hopkins were recruited both prospectively and retrospectively, with the prospectively recruited group having a larger proportion of autoimmune encephalitis. This may be due to the prospective cohort being done more recently, as there is more clinician familiarity with the autoimmune encephalitides as well as improved autoantibody testing. Reassuringly, the 2 different recruitment methods produced groups similar in all other demographic measures, including illness severity and causes of immunocompromised status.

The focus of our study on encephalitis as a syndrome, as well as the subanalyses conducted on the infectious and autoimmune classes, gives broader clinical utility to our study as compared with those focusing on specific etiologies. The study's retrospective nature is its most significant limitation, as the data collected were limited to what was readily available in the medical record. Thus, immunocompromised status was broadly defined, as variables such as CD4 count at the time of hospitalization were not readily available for many patients. Additionally, as VZV and CMV can be positive from CSF even if not the cause of encephalitis, it is possible that some of these cases could represent incidental reactivation. We are unable to determine causality between features such as clinical presentation, diagnostic studies, treatments, and outcome and can only discuss correlations seen in the data, which may have unexpected confounders.

## CONCLUSIONS

A substantial proportion of patients with encephalitis are immunocompromised. These patients are at higher risk for many opportunistic infections, and outcomes are worse than in immunocompetent individuals. Notably, HSV encephalitis accounts for a similar proportion of cases in both groups, although CSF neutrophilia, perhaps because of impaired lymphocyte responses, occurs more commonly in the immunocompromised group. Surprisingly, autoimmune encephalitis accounts for 10% of cases of encephalitis in the immunocompromised and is most commonly due to the use of checkpoint inhibitors. Prognostic factors differ substantially between immunocompetent and immunocompromised individuals with encephalitis. Most notably, autoimmune encephalitis portends a poorer in-hospital prognosis in immunocompetent individuals. The more benign course of autoimmune encephalitis in immunocompromised individuals is likely a function of the reversibility of checkpoint inhibitor–associated encephalitis, along with the natural history of autoantibody-associated encephalitis when the immune system is compromised. Knowledge of the spectrum of etiologies, clinical presentations, and prognosis in encephalitis in the immunocompromised can inform the diagnosis and management of these patients, even as the epidemiology continues to change in this era of increased adoption of targeted immunocompromising agents for rheumatologic and neurologic disorders, checkpoint inhibitors and CAR-T-cell-based therapies for malignancies, and increased recognition of infection-associated autoimmune encephalitides.

## Supplementary Material

ofaf332_Supplementary_Data

## Supplementary Data

Supplementary materials are available at *Open Forum Infectious Diseases* online. Consisting of data provided by the authors to benefit the reader, the posted materials are not copyedited and are the sole responsibility of the authors, so questions or comments should be addressed to the corresponding author.
