# Supplementary material for: Encephalitis in Immunocompromised vs Immunocompetent Patients: A Comparative Study
Source: Open Forum Infect Dis. 2025 Jun 11;12(7):ofaf332. doi: 10.1093/ofid/ofaf332 (PMC12216899; doi:10.1093/ofid/ofaf332)
Supplement: ofaf332_Supplementary_Data [file ofaf332_supplementary_data.docx]

Supplementary Table 1: Causes of Immunocompromised Status in Entire Cohort

| Causes of Immunocompromised Status | Number of Patients |
| --- | --- |
| HIV | 59 (39%) |
| Active Malignancy | 39 (25%) |
| Solid Organ or Bone Marrow Transplant | 9 (6%) |
| >20 mg prednisone daily or equivalent for >1 month | 46 (30%) |

Abbreviations: HIV: Human Immunodeficiency Virus, mg: milligrams

Supplementary Table 2: Demographics, Clinical and Lab Findings, and Outcome Variables for Infectious Encephalitis Patients

|  | Immunocompromised | | P value |
| --- | --- | --- | --- |
|  | Yes | No |  |
| Patients, # | 85 | 183 |  |
| Sex |  |  | 0.337 |
| Female | 49 | 94 |  |
| Male | 36 | 89 |  |
|  |  |  |  |
| Mean Age of Onset (SD) | 49.99 (14.2) | 53.30 (16.3) | 0.093 |
| Total CCI> 2 | 70/85 (82%) | 76/183 (42%) | **<0.001***** |
|  |  |  |  |
| **Clinical and Lab Findings** |  |  |  |
| Fever | 49/80 (61%) | 132/182 (73%) | 0.069 |
| New Focal CNS Findings | 40/85 (47%) | 60/183 (33%) | **0.025*** |
| Seizures | 22/84 (26%) | 53/183 (29%) | 0.640 |
| Headaches | 42/77 (55%) | 105/166 (63%) | 0.196 |
| Sleep Disturbance | 12/73 (16%) | 16/178 (9%) | 0.089 |
| Memory Loss | 12/78 (15%) | 32/181 (18%) | 0.652 |
| Abnormal Movements | 10/80 (13%) | 16/182 (9%) | 0.355 |
| CSF WBC ≥ 5/mm^3^ | 51/81 (63%) | 134/179 (75%) | **0.05*** |
|  |  |  |  |
| CSF WBC Neutrophils % > 50 | 17/74 (23%) | 39/157 (25%) | 0.757 |
| CSF Protein < 50 mg/dL | 16/83 (19%) | 27/179 (15%) | 0.394 |
| CSF Glucose < 45 mg/dL | 36/85 (42%) | 35/180 (19%) | **<0.001***** |
|  |  |  |  |
| Peripheral WBC > 11K/ µL | 23/84 (27%) | 63/180 (35%) | 0.219 |
| MRI Abnormalities | 49/67 (74%) | 91/131 (69%) | 0.591 |
| Abnormal EEG | 40/45 (89%) | 76/95 (80%) | 0.192 |
| Glasgow Coma Scale (median, IQR) | 14.00 (3.00) | 14.00 (3.00) | 0.337 |
| Subacute to Chronic Onset | 18/79 (23%) | 39/179 (22%) | 0.859 |
|  |  |  |  |
| **Outcomes** |  |  |  |
| Hospital Length of Stay (mean, SD) | 22.73 (27.8) | 14.14 (15.6) | **0.01**** |
| Glasgow Outcome Scale < 4 | 56/78 (72%) | 67/165 (41%) | **<0.001***** |
| Death | 11/85 (13%) | 12/183 (7%) | 0.083 |

Abbreviations: SD: standard deviation, CCI: Charlson comorbidity index, CNS: central nervous system, mg: milligrams, dL: deciliter, mm: millimeter, K: thousand, MRI: magnetic resonance imaging, IQR: interquartile range. Asterisks indicate levels of significance: p < 0.05 (*), p < 0.01 (**), and p < 0.001 (***).

Supplementary Table 3: Glasgow Outcome Scale Predictor Variables, by Sub analysis Group

*Cohort a: Infectious Encephalitis, Immunocompromised*

| Significant Predictors | Glasgow Outcome Scale | | P value |
| --- | --- | --- | --- |
|  | GOS < 4 | GOS ≥ 4 |  |
| Age > 60 | 20/56 (36%) | 2/22 (9%) | 0.019 |
| CSF Neutrophils% > 50 | 15/51 (29%) | 1/18(6%) | 0.039 |
| CSF Glucose < 45 mg/dL | 20/56 (36%) | 14/22 (64%) | <0.001 |

*Cohort b: Infectious Encephalitis, Non- Immunocompromised*

| Significant Predictors | Glasgow Outcome Scale | | P value |
| --- | --- | --- | --- |
|  | GOS < 4 | GOS ≥ 4 |  |
| Age > 60 | 37/67 (55%) | 27/98 (28%) | <0.001 |
| New Focal CNS Findings at Onset | 26/67 (39%) | 24/98 (24%) | 0.049 |
| Inpatient Seizures | 15/67 (22%) | 7/98 (7%) | 0.005 |
| Headaches at onset | 30/57 (53%) | 67/94 (71%) | 0.02 |
| Memory Loss at Onset | 20/67 (30%) | 11/98 (11%) | 0.003 |
| Abnormal Movements at Onset | 12/66 (18%) | 2/98 (2%) | <0.001 |
| Peripheral WBC > 11K/ µL | 30/67 (45%) | 28/97 (29%) | 0.036 |

*Cohort c: HSV Encephalitis, Immunocompromised*

| Significant Predictors | Glasgow Outcome Scale | | P value |
| --- | --- | --- | --- |
|  | GOS < 4 | GOS ≥ 4 |  |
| Charles Comorbidity Index < 2 | 1/21 (5%) | 2/4 (50%) | 0.011 |

*Cohort d: HSV Encephalitis, Non-Immunocompromised*

| Significant Predictors | Glasgow Outcome Scale | | P value |
| --- | --- | --- | --- |
|  | GOS < 4 | GOS ≥ 4 |  |
| New Focal CNS Findings at Onset | 11/23 (48%) | 6/34 (18%) | 0.015 |
| Abnormal Movements at Onset | 6/22 (27%) | 1/34 (3%) | 0.007 |
| Peripheral WBC > 11K/ µL | 7/23 (30%) | 2/33 (6%) | 0.015 |

Abbreviations: SD: standard deviation, CCI: Charlson comorbidity index, CNS: central nervous system, mg: milligrams, dL: deciliter, mm: millimeter, K: thousand, MRI: magnetic resonance imaging, IQR: interquartile range. Asterisks indicate levels of significance: p < 0.05 (*), p < 0.01 (**), and p < 0.001 (***).
